# Peer education strategies validated through Delphi at an urban university in KwaZulu-Natal

**DOI:** 10.4102/hsag.v30i0.3127

**Published:** 2025-09-30

**Authors:** Thobile I. Zulu, Robert T. Netangaheni

**Affiliations:** 1 Department of Sociology, College of Human Sciences, University of South Africa, Pretoria, South Africa; 2 Department of Health Studies, College of Human Sciences, University of South Africa, Pretoria, South Africa

**Keywords:** peer education, Delphi method, higher education, strategy validation, phenomenography, consensus-building, strategy development

## Abstract

**Background:**

Peer education is vital in higher education because it promotes behavioural change, human immunodeficiency virus (HIV) prevention, student wellness and academic success. However, limited research exists on the expert validation of such strategies, particularly within higher education institutions. This study addresses this gap by validating peer education strategies using a modified Delphi method.

**Aim:**

This study aimed to validate strategies for peer education in higher education institutions through expert consensus using the Delphi method.

**Setting:**

The study was conducted across South African higher education institutions, engaging stakeholders in student wellness and peer-led programmes.

**Methods:**

A two-round modified Delphi method was employed to validate peer education strategies developed through a multiphased qualitative design. The strategies were informed by an integrative literature review and phenomenographic inquiry capturing the lived experiences of peer educators. A panel of 10 purposively selected experts from diverse higher education institutions evaluated the strategies through structured questionnaires using a five-point Likert scale. Consensus was defined as ≥ 70% agreement per item.

**Results:**

Consensus was achieved across all strategic domains, including scope, evidence-based design, stakeholder inclusivity, clarity, applicability and editorial independence. Based on expert feedback, the strategies were refined to improve their practical applicability while ensuring stronger alignment with institutional structures and priorities.

**Conclusion:**

The validated strategies provide a systematic, context-specific guide for the design and implementation of peer education programmes in higher education. They emphasise adaptability, stakeholder collaboration and institutional alignment to support programme sustainability and student well-being.

**Contribution:**

This study offers validated strategies to strengthen the effectiveness, quality and impact of peer-led interventions in higher education.

## Introduction

Institutions of Higher Learning are increasingly relying on peer education as a strategic approach to support behavioural change interventions, promote human immunodeficiency virus (HIV) prevention, and enhance student development, wellness, and academic success. Peer education programmes harness the power of students educating their peers, enabling the transfer of knowledge, the development of leadership skills and the provision of psychosocial support within the academic environment. These programmes are particularly effective in addressing sensitive issues, such as sexual health, substance abuse and mental health, where students may feel more comfortable engaging with peers than with authority figures.

While peer education programmes hold considerable promise in fostering positive behavioural and attitudinal changes among adolescents, research indicates that their implementation often varies across contexts. This variability is partly because of the limited availability of validated strategies that can consistently inform their design, execution and evaluation (Chowdhary et al. 2022). In some cases, these programmes are introduced with minimal institutional support, limited long-term sustainability planning, and underdeveloped monitoring and evaluation mechanisms, which can affect their overall impact and scalability (Mayanja 2020; Nyarayi & Sibanda 2021). Furthermore, the absence of standardised models and integration within institutional structures contributes to differences in programme structure and delivery, potentially influencing their effectiveness in achieving desired outcomes (Chowdhary et al. 2022).

Research from various contexts highlights the positive impact of peer education. For example, a randomised controlled trial conducted at a Malaysian public university assessed the effects of peer-led education on students’ knowledge, attitudes and behaviours related to HIV (Ibrahim et al. 2012). The study found that participants in the peer-led programme demonstrated significant improvements in HIV-related knowledge and attitudes, along with a reduction in substance-related risk behaviours. However, it also revealed limited changes in sexual risk behaviours, indicating that peer education strategies must be tailored and targeted to effectively address complex behavioural change domains. These findings highlight the need for structured, evidence-based frameworks that can enhance the relevance and adaptability of peer education programmes.

To this end, this study aims to bridge the gap between theory and practice by developing and validating strategies for peer education within higher education. The study builds on earlier research that employed phenomenography to explore the diverse experiences of peer educators. This approach revealed nuanced insights into how students perceive and enact their roles, highlighting the importance of context, identity and institutional culture in shaping programme outcomes.

To ensure credibility and practical value of the strategies developed, this study employed the Delphi method, which involved a structured, consensus-driven approach that gathered expert opinions through iterative rounds of evaluation and refinement. Combining qualitative insights with expert consensus enhances the proposed strategies’ depth and applicability.

### Aim

This study used the Delphi method to develop and validate peer education strategies through expert consensus.

#### Study objectives

- To validate the proposed strategies through a Delphi process involving experts in health promotion and higher education.
- To establish consensus-based recommendations for the effective implementation and institutionalisation of peer education programmes in diverse academic settings.

## Research methods and design

### Study design

This study employed a mixed qualitative approach, incorporating phenomenography, an integrative literature review, and the Delphi technique to develop and validate a peer education strategy for an urban-based university. Phenomenography was used to explore peer educators’ varying experiences and perceptions, offering nuanced insights into their roles and challenges (Masava, Nyoni & Botma 2023a). These findings were synthesised with an integrative literature review to inform strategy development, ensuring the framework was both evidence-based, and contextually relevant.

The first phase consisted of a comprehensive literature review aimed at synthesising current scholarly and empirical research on peer education within higher education. This laid the groundwork for pinpointing essential success factors, contextual elements and challenges related to the implementation of peer education programmes (Schulte 2020; Zdunek, Strecker & Sandner 2020).

The second phase employed a phenomenographic approach to examine the diverse conceptions and experiences of peer education among peer educators. This qualitative method allowed the researcher to identify unique categories of description, which represent various understandings of the phenomenon (Masava et al. 2023b). The results from this phase were compared with the literature review to guide the creation of evidence-based, context-specific strategies.

In the third phase, a modified Delphi technique was employed to validate the proposed strategies. A purposive sample of 10 experts was recruited from various institutions of higher learning, including programme managers, health promoters and academic heads with practical involvement in peer education (Linhares et al. 2019). The Delphi process involved two rounds of data collection using structured questionnaires, comprising 24 statements organised into six strategic domains. A five-point Likert scale was used to rate the relevance and applicability of each statement (Al-Qawasmi 2021; Vogel et al. 2019).

In Round 1, expert feedback was collected, and areas lacking consensus were revised based on the panel’s qualitative input. Minor refinements, such as rewording ‘framework’ to ‘strategies’, were incorporated into the second round. Round 2 confirmed full consensus across all domains, including scope and purpose, rigour, stakeholder involvement, clarity and presentation, applicability, and editorial independence. Consensus was defined as ≥ 70% of panel members indicating agreement or strong agreement with each item (Morris 2024).

This method was deemed particularly suitable for this study because of the limited availability of established frameworks for peer education strategies in higher education. Furthermore, the Delphi approach mitigates the potential influence of the researcher’s personal bias by prioritising expert perspectives gathered independently and anonymously through multiple feedback rounds (Junger et al. 2023). The method is also logistically effective when participants are geographically dispersed, as it allows for remote, asynchronous participation, which is an important consideration for this study, where the expert panel included members from various institutions and regions (Keeney, McKenna & Hasson 2024; Vogel et al. 2022).

Furthermore, evidence from prior studies indicates that the Delphi technique has gained increasing recognition in recent years as a robust methodological tool for developing evidence-informed frameworks and facilitating expert consensus within the domains of health education and policy formulation. Its methodological flexibility, combined with its capacity to systematically capture and synthesise input from a heterogeneous panel of experts, has positioned it as a method of choice, particularly in interdisciplinary and international research contexts where in-person engagement is logistically challenging (Chan, Wong & Cheung 2020; Hasson & Keeney 2021; Szeto et al. 2024). In the context of this study, the Delphi technique was deemed the most appropriate approach for validating the developed peer education implementation strategies. This decision was further justified by the feasibility of conducting the process electronically via email correspondence, which afforded experts the flexibility to participate asynchronously, thereby accommodating their schedules and availability.

The study adhered to established principles of trustworthiness to ensure methodological integrity. Credibility was upheld through the purposive selection of experts, while confirmability was reinforced by grounding the analysis in empirical data. Dependability was achieved through the consistent application of a systematic research design, and transferability was supported by providing rich, contextual descriptions to facilitate application in comparable settings (Amiri 2024; Carolan et al. 2022; Kadıoglu et al. 2021; Helms, Gardner & McInnes 2016). Anonymity was maintained throughout the Delphi rounds to mitigate potential bias and reduce the influence of dominant voices, enabling equal participation. The iterative nature of the Delphi method further allowed experts to reflect and refine their perspectives across successive rounds (Jacob, Duffield & Jacob 2018). This rigorous, transparent and participatory approach ensured that the validated peer education strategies are not only evidence-informed but also contextually appropriate and institutionally sustainable for implementation in higher education.

### Development of strategies

The development of the initial strategies was informed by a triangulated synthesis of phenomenographic data and an integrative literature review. This triangulation was intentionally employed to enhance both the theoretical robustness and practical relevance of the proposed strategies, thereby aligning with established best practices in strategy development (Schulte 2020; Zdunek et al. 2020). The resultant strategies were categorised across six key domains: scope and purpose, rigour, stakeholder involvement, clarity and presentation, applicability, and editorial independence. The Delphi study constituted a core component of the broader doctoral research and was designed as a structured, multiphase process to refine and validate the strategies intended to shape a peer education programme. The initial phase involved the implementation of an integrative literature review, conducted systematically per Whittemore and Knafl’s (2005) five-step framework. This facilitated a comprehensive synthesis of existing scholarly evidence, enabling the identification of knowledge gaps related to peer education within higher education settings (Cho 2022). Subsequently, qualitative data were gathered through semi-structured, face-to-face interviews with 20 peer educators from an urban-based university spanning two districts in KwaZulu-Natal. Employing a phenomenographic methodology enabled an in-depth exploration of participants’ varying conceptions and lived experiences within the peer education context (Marton 1981; Yates, Partridge & Bruce 2012), thereby enriching the empirical foundation upon which the initial strategies were constructed.

Findings from the literature review and qualitative interviews were triangulated to formulate preliminary strategies, a process that enhanced the rigour and credibility of the findings (Kettunen & Tynjala 2018). These were subsequently validated using a modified Delphi technique, which engaged a purposively selected panel of experts with practical and managerial experience in peer education (Green 2014; Okoli & Pawlowski 2004). The Delphi process was conducted in two iterative rounds, incorporating structured questionnaires and anonymous feedback to reach consensus (Hsu & Sandford 2007; Senerth et al. 2025). This approach not only enhanced the credibility and contextual relevance of the strategies but also ensured inclusivity by enabling asynchronous expert participation across geographic locations (Linstone & Turoff 2020).

### Phase 1: Qualitative research with peer educators

The study began with qualitative research employing a phenomenographic approach to explore the qualitatively distinct ways in which peer educators at an urban-based university in KwaZulu-Natal experience and conceptualise their roles within the peer education programme. The findings from Phase 1 of the study were derived through in-depth, semi-structured interviews with 20 peer educators, and the analysis followed a rigorous seven-step process, supported by ATLAS.ti software. The aim was to uncover collective conceptions rather than individual opinions.

The data analysis yielded seven categories of description, each representing a qualitatively unique way in which peer educators understood and experienced their involvement in the programme. These categories, empowerment through participation, transformation and personal growth, challenges faced, support and supervision, impact on peers, the need for improvement, and relational engagement, formed the core thematic foundation for strategy development. Central to the phenomenographic approach is a relational and non-dualistic perspective, and the categories reflected both individual and collective dimensions of meaning-making among peer educators.

These seven categories were synthesised through the iterative process into an ‘outcome space’, representing a structured and hierarchical framework of the peer educators’ experiences. This space illustrates a progression from basic engagement to deeply reflective and transformative understandings of the peer education programme. The outcome space serves as a conceptual map for informing strategy development and institutional support mechanisms. The findings provided critical insights into how peer educators navigate and interpret their roles, offering a rich basis for informing strategic interventions aimed at enhancing peer education in higher education contexts. Specifically, the outcome space informed the development of strategies focused on:

- Strengthening institutional support and recognition.
- Tailoring training and mentorship programmes.
- Embedding peer education within university health and wellness policies.
- Promoting inclusive and transformative peer-led initiatives.

These qualitative insights from Phase 1 directly influenced the formulation of strategies in Phase 2 and were subsequently validated through the Delphi method in Phase 3. Overall, the study highlights the transformative and relational nature of peer education and demonstrates its potential as a foundational component in student support and development strategies within university settings.

### Phase 2: Integrative literature review

In addition to the qualitative research, an integrative literature review was also conducted to develop strategies that would be validated using the Delphi method. This review was part of a triangulated approach, along with qualitative data analysis from Phase 1 of the study. The purpose of the review was to synthesise existing literature on peer education, particularly from a phenomenographic perspective, to identify strategic opportunities for innovation and improvement in current programme practices.

The review followed the seven-step framework proposed by Whittemore and Knafl (2005), enabling a systematic process for identifying, evaluating, and synthesising diverse sources. The literature search was conducted between 01 September and 11 October 2024, using multiple academic databases, including Google Scholar, Summons, open-access repositories, and ProQuest Dissertations. The search strategy targeted peer-reviewed articles published between 2014 and 2024, written in English, and focused on peer education strategies in higher education. Specific keywords included ‘phenomenography’, ‘peer education strategies’, ‘peer educators’, and ‘higher education’.

The identified studies were critically appraised using the Critical Appraisal Skills Programme (CASP) qualitative checklist, ensuring methodological rigour and relevance. Out of 150 records initially retrieved, 49 studies met the inclusion criteria. Of these, 29 were synthesised thematically for in-depth analysis. The selected studies represented a broad geographical spread, including countries such as South Africa, Turkey, Sweden, Rwanda, Australia, China, and Thailand, contributing a rich global perspective to the review.

The findings were categorised into five overarching thematic areas that directly informed strategy development: Personal and Professional Development. Several studies, including Southgate and Angleton (2017a); Weston (2018a); Nkurunziza et al. (2024b), highlighted the role of peer education in building leadership, communication, and confidence among peer educators. Training programmes were recommended to include mentorship, interpersonal skills, and self-reflection practices to enhance peer educators’ effectiveness and personal growth:

- Challenges in Programme Implementation: Common challenges included recruitment inefficiencies, ethical dilemmas, and inconsistencies in programme delivery. Authors such as Nygren and Carlson (2017) and Gobbo, Russo and Bellini (2023) advocated for structured recruitment processes, ethical training modules, and fidelity to programme design to ensure sustainable impact.
- Engagement Strategies and Digital Tools: Innovative delivery methods, including digital messaging and online engagement platforms, were shown to improve accessibility and interaction (Choorat et al. 2018; Salzman 2014; Zhao 2024). These tools aligned with evolving student communication preferences and promoted broader outreach.
- Long-Term Outcomes and Community Impact: Peer education has been increasingly recognised as a catalyst for sustained personal development and broader community transformation. Empirical research by Frawley and Bigby (2014) and Choorat et al. (2018) highlights the capacity of peer-led initiatives to foster meaningful community engagement, empower participants, and stimulate behavioural change that extends beyond the confines of formal educational settings. Specifically, programmes that integrate self-advocacy within peer support structures are not only instrumental in enhancing student empowerment but also play a critical role in addressing systemic barriers that hinder academic success. Evidence further indicates that these initiatives help cultivate inclusive and supportive learning environments through the strengthening of interpersonal relationships among students, faculty, and institutional staff (Beard, Schilt & Jagoda 2023). Such peer-led frameworks have been shown to promote increased self-confidence and academic participation, particularly among students with disabilities, thereby corroborating the findings of Frawley and Bigby (2014) and extending them through subsequent studies such as Shiyanbola, Brown and Ward (2018).
- Educational Transitions: Research by Henttonen, Niemi and Rantanen (2022), Salzman (2014), and Zhao (2024) highlighted the role of peer educators in supporting students during transitions into higher education, particularly through emotional support, academic guidance, and the development of social networks.

The integrative review provided a comprehensive synthesis of evidence-based strategies that guided the design of a peer education framework suitable for urban-based university contexts. The triangulated approach, combining literature insights with empirical qualitative data, allowed for the formulation of practical and context-sensitive strategies aimed at improving peer education effectiveness, scalability, and inclusivity.

### Phase 3: Validation process: The Delphi technique

To validate the proposed strategies, the study employed a modified Delphi method, which is effective in achieving expert consensus through structured, iterative rounds (Carolan et al. 2022; Linhares et al. 2019). The method was chosen for its capacity to elicit anonymous, unbiased feedback from professionals with extensive experience in peer education (Kadıoglu et al. 2021). According to Jacob et al. (2018), the Delphi technique also enables exploration of complex issues, allowing strategies to be refined through expert input:

- *Round 1*: Experts received brief background information on the formulation of the strategies and used a five-point scale to rate 24 items, indicating their level of agreement. Open-ended comments were also solicited for qualitative insight.
- *Round 2*: Feedback from Round 1 led to minor modifications – most notably, replacing the term ‘framework’ with ‘strategies’ for clarity. Revised statements were presented, and consensus was achieved across all six domains (Nowak et al. 2019).

Consensus was defined as at least 70% of participants agreeing or strongly agreeing on each item, a threshold consistent with accepted Delphi study standards (Morris 2024).

## Draft strategies shaping the peer education programme

The formulation of the proposed strategies for peer education was guided by a systematic process rooted in both empirical and theoretical insights. Similar to the Delphi guide developed by Bentley, Mantzicopoulos and Sahin (2023) to implement interprofessional education (IPE) in primary healthcare settings, the current study employed a phased approach to develop and refine peer education strategies within higher education. While Bentley et al.’s framework was initially intended for IPE in clinical and community placements, its applicability to the development of structured educational strategies informed the methodological rigour of this study (Bentley et al. 2023).

Drawing from the triangulated findings of a phenomenographic investigation and an integrative literature review, the researcher developed an initial set of strategies tailored to the contextual realities of higher education. This preliminary draft was then refined before expert engagement, echoing Bentley et al.’s (2023) approach of refining frameworks before initiating the consensus-building process. In line with their model, the draft strategies were categorised into key domains relevant to peer education, such as the programme’s scope and purpose, stakeholder engagement, clarity of roles, practical implementation, and editorial independence (Bentley et al. 2023).

The strategies encompassed core areas, including the relevance of peer education in addressing student wellness, institutional and organisational readiness, cultural considerations, required competencies for peer educators, pedagogical methods, assessment mechanisms, modes of delivery and public health concerns. Similar to Bentley et al.’s adaptation of coronavirus disease 2019 (COVID-19) precautions into broader public health considerations over multiple Delphi rounds, this study also evolved its constructs iteratively, incorporating expert feedback to ensure relevance and responsiveness to the higher education context (Bentley et al. 2023).

A structured Delphi process with two iterative rounds allowed the panel of experts to assess and refine the proposed strategies using a five-point Likert scale. This method facilitated expert consensus on 24 strategy statements across six domains, resulting in the validation of robust and contextually grounded strategies for peer education programmes in higher education (Masava et al. 2023a; Nawagi et al. 2023).

### Setting

The study was conducted across selected South African Institutions of Higher Learning, specifically those engaged in implementing peer education focusing on HIV prevention, student support services and the government agencies collaborating with the institutions of higher learning. These institutions reflect a diverse landscape, varying in geographic location, institutional focus and student population profiles. Within these settings, peer education programmes are commonly employed to foster health awareness, encourage positive behavioural change and support academic achievement. However, the implementation of such programmes remains inconsistent and fragmented. The distinctive context of South African higher education, which is characterised by both significant challenges and unique opportunities, offered a meaningful and dynamic setting for this research.

### Study population and sampling strategy

The study population consisted of experts in peer education and health promotion who were either employed within or collaborated with institutions of higher learning. To be included in the study, participants were required to have at least 5 years of experience in the design, implementation or management of peer education programmes.

A purposive sampling strategy was employed to identify a pool of 20 potential experts from universities, health organisations and government-linked education agencies. From this pool, 10 participants agreed to participate and completed both Delphi rounds. The panel included a diverse mix of professionals such as programme managers, health promoters, student support coordinators and department heads, ensuring a range of institutional and professional perspectives.

Table 1 outlines the institutional affiliations and specific areas of expertise of the experts who contributed to the study.

**TABLE 1:** Frequency distribution of the characteristics of the Delphi panel experts, *N* = 10.

Variable	Frequency (*N*)
**Gender**	
Male	6
Female	4
**Discipline**	
Health promotion	4
Social science	5
Nursing	1
**Institutional affiliation**	
Durban University of Technology	2
University of Zululand	2
Mangosuthu University of Technology	2
Higher Health	1
University of KwaZulu-Natal	1
Drama in Aids Education (DramAidE)	1
Department of Health	1
**Expertise**	
Programme management	2
Health promotion and peer education supervision	5
Academic leadership	3

### Sample size

While no formal sample size calculation was employed, the Delphi method does not necessitate large samples; the depth of expertise remains paramount. The selected panel size complies with established guidelines for Delphi studies, which recommend 10–18 participants for effective consensus building (Hasson, Keeney & McKenna 2000; Mukherjee, Mantzicopoulos & Sahin 2023), also informed by the homogeneity of the background of the experts selected (Hsu & Sandford 2007).

As Nawagi et al. (2023) asserts, the Delphi method allows researchers to use their discretion in determining an appropriate panel size based on the scope of the problem and available resources. It is crucial to carefully consider the panel’s collective expertise, the diversity of perspectives, and the relevance of the participants’ experience to the construct under investigation. Following these methodological principles, 10 experts were intentionally selected for this study. Although this number is smaller than some conventional recommendations, it is well within the documented range for Delphi panels, which can vary from 8 to 1685 participants, depending on the study’s aims and context (Nawagi et al. 2023). Thus, the decision was driven by the quality and relevance of expert knowledge rather than quantity, ensuring meaningful contributions to the consensus-building process.

### Data collection

Data were collected using structured questionnaires distributed electronically during two Delphi rounds. The questionnaire utilised a five-point Likert scale (ranging from ‘strongly disagree’ to ‘strongly agree’) to measure expert agreement on each framework component. Open-ended sections allowed experts to provide qualitative feedback and recommend modifications to improve clarity, relevance or feasibility. The questionnaire was piloted before distribution to ensure clarity and relevance.

In practical terms, the surveys were administered via email. Experts were given clear instructions, confidentiality assurances and sufficient time (10 days–14 days) to respond. Follow-up reminders were sent to ensure maximum participation. No significant issues such as language barriers or technological constraints were encountered. The use of electronic surveys facilitated efficient data collection and allowed experts from various geographic locations to participate (Hsu & Sandford 2007).

### Data analysis

Quantitative responses were collated and analysed using descriptive statistics to assess levels of agreement for each framework domain. Consensus was defined as 70% or more of participants selecting either ‘agree’ or ‘strongly agree’. Areas falling below this threshold were revised based on qualitative comments and reassessed in the next round.

In this study, qualitative feedback underwent a thematic analysis to discern patterns and trends within the responses. The data collected were systematically categorised and coded, enabling the identification of prevalent suggestions and concerns that subsequently guided modifications to the framework in question. The data cleaning phase involved meticulous verification for any missing responses or discrepancies between the quantitative Likert scale ratings and the qualitative comments provided by participants. This rigorous process ensured that the ultimate decisions regarding revisions to the framework were grounded in empirical evidence and reflective of a consensus among experts in the field. By integrating both quantitative and qualitative analyses, the study afforded a comprehensive understanding of expert insights, thereby facilitating the development of a robust and well-informed framework (Nowell et al. 2023). As the table presents the outcomes of Round 2, the column “Pursue to Round 2” used in Round 1 to indicate progression of items is no longer applicable. In Round 2, the focus was on final validation and consensus-building rather than advancing items to subsequent rounds. Accordingly, this column was removed to enhance clarity and to ensure the table reflects the definitive outcomes of the Delphi process.

### Ethical considerations

Ethical clearance was obtained from the University of South Africa’s College of Human Sciences Research Ethics Review Committee (Ref: 67132170_crec_chs_2023), and all participants provided informed consent before participation. Participant anonymity was ensured, and no individual was provided access to information about other participants involved in the study.

## Results

### Demographic characteristics of experts

The expert panel consisted of 10 participants (3 male and 7 female), aged 28 years – 59 years. Their positions included Heads of Departments, programme managers and health promoters. They possessed varied qualifications, from diplomas to master’s degrees, and had experience ranging from 5 to 25 years in peer education.

### Results of Round 1

Engagement with the expert panel was facilitated through email correspondence, a necessary approach given the participants’ diverse geographical distribution. The Delphi process was conducted over two iterative rounds. Initial stakeholder engagement involved assembling a purposively selected panel of experts and initiating contact via an introductory email. This communication provided a comprehensive overview of the study’s objectives, the expected participation process, and the timelines associated with each Delphi round. The use of email communication served to establish rapport and secure expert commitment to the process. All data collection activities were administered through Google Forms, an online survey tool that enabled efficient distribution of the instrument and streamlined the aggregation and preliminary analysis of responses for the research team.

In Round 1 (Table 2), a structured questionnaire comprising 24 statements under six thematic domains was used to solicit expert input. These domains included: (1) Scope and Purpose, (2) Rigour, (3) Stakeholder Involvement, (4) Clarity and Presentation, (5) Applicability, and (6) Editorial Independence. Each item was rated using a five-point Likert scale (strongly disagree, disagree, neutral, agree, strongly agree), and participants were invited to comment on each statement.

**TABLE 2:** Round 1 consensus on the strategies of peer education programme statements *N* = 10.

Domain	Questions	Mode of consensus	Intensity of consensus (%)	General positive or Negative consensus (%)	Remarks	Pursue to Round 2
1. Scope and purpose	The overall purpose of the strategies for peer education strategies is clearly defined.	Strongly Agree	100	100	Consensus achieved	No
	The strategies are aligned with institutional goals.	Strongly agree	100	100	Consensus achieved	No
2. Rigour	The strategies’ objectives reflect the target audience’s priorities (students and peer educators).	Strongly agree	100	100	Consensus achieved	No
	Evidence-based practices are incorporated into the framework’s design.	Strongly agree	90	90	Consensus achieved	No
3. Stakeholder involvement	The framework addresses the needs of the marginalised student population.	Strongly agree	90	90	Consensus achieved	No
	The framework includes all the stakeholders.	Strongly agree	100	100	Consensus achieved	No
4. Clarity and presentation	Roles are clearly articulated in the framework.	Strongly agree	90	100	Consensus achieved	No
5. Applicability	The suggested strategies can be practically implemented in the context of higher education.	Strongly agree	100	90	Consensus achieved	No
	The model can be adapted to diverse institutional or community contexts.	Agree	100	100	Consensus achieved	No
	Strategies are relevant to current challenges faced by students and peer educators.	Strongly Agree	100	100	Consensus achieved	No
6. Editorial independence	The framework balances stakeholder input with evidence-based practices.	Agree	50	70	Consensus partially achieved	Yes
	The framework maintains objectivity in its content and recommendations.	Agree	60	70	Consensus partially achieved	Yes
	The framework is free from potential biases, conflicts of interest, or undue influence from external parties.	Strongly agree	100	100	Consensus achieved	Yes

There is no universally accepted standard for defining consensus in Delphi studies worldwide. Researchers applied context-specific methods to determine consensus-based study design and objectives. In this study, the approach was guided by the work of Niederberger and Spranger (2020), who recommend using a percentage level of agreement to measure consensus in health-related Delphi research. Accordingly, a 70% threshold or more agreement (i.e., ‘agree’ or ‘strongly agree’) on a given item was deemed indicative of consensus, while items receiving 69% agreement or less were designated for further deliberation in the subsequent round.

In this initial round, there was strong consensus across most domains. The domain *Scope and Purpose* achieved full consensus (100%) on all statements, indicating clarity and alignment of the strategies with institutional goals. The *Rigour* domain also received high agreement – 100% of participants agreed that the objectives reflected audience priorities, while 90% confirmed the integration of evidence-based practices.

In terms of stakeholder involvement, most participants demonstrated a strong level of agreement (90%–100%), indicating that the strategies effectively addressed the specific needs of marginalised student populations and ensured the inclusion of all pertinent stakeholders. Similarly, the dimensions of clarity of presentation and applicability received unanimous endorsement across all measured items. This consensus suggests that the strategies not only articulated stakeholder roles with clarity but also presented strategies that are sufficiently flexible to be adapted across a range of institutional and community contexts.

However, the domain of editorial independence demonstrated only partial consensus, with levels of agreement ranging from 50% to 70% across specific items. Participants expressed particular uncertainty regarding the extent to which the framework maintained a balance between stakeholder input and objective, evidence-based decision-making. Notably, two experts selected the ‘neutral’ response option, citing ambiguity around the term ‘framework’ as it was presented in the assessment items. Considering this feedback, and to improve conceptual clarity, the terminology was refined in subsequent rounds, replacing ‘framework’ with ‘strategies’, to accurately reflect the practical components under evaluation and to facilitate clearer interpretation among participants.

Overall, Round 1 successfully established consensus on the majority of the strategy domains, laying a strong foundation for refining statements that lacked sufficient agreement. The structured use of Likert-scale responses, combined with expert commentary, proved effective in identifying areas requiring clarification and strengthening to be addressed in Round 2 (Table 3).

**TABLE 3:** A summary of Round 2 results.

Domain	Questions	Mode of consensus	Intensity of consensus (%)	General positive or Negative consensus (%)	Remarks
1. Scope and purpose	The overall purpose of the strategies for peer education strategies is clearly defined.	Strongly agree	100	100	Consensus achieved
	The strategies are aligned with institutional goals.	Strongly agree	100	100	Consensus achieved
2. Rigour	The objectives of the strategies reflect the priorities of the target audience (students and peer educators).	Strongly agree	100	100	Consensus achieved
	Evidence-based practices are incorporated into the framework’s design.	Strongly agree	90	90	Consensus achieved
3. Stakeholder involvement	The strategies address the needs of the marginalised student population.	Strongly agree	90	100	Consensus achieved
	The strategies are inclusive of all the stakeholders.	Strongly agree	100	100	Consensus achieved
4. Clarity and presentation	Roles are clearly articulated in the strategies.	Strongly agree	90	100	Consensus achieved
5. Applicability	The suggested strategies can be practically implemented in the context of higher education.	Strongly agree	100	90	Consensus achieved
	The model can be adapted to diverse institutional or community contexts.	Agree	100	100	Consensus achieved
	Strategies are relevant to current challenges faced by students and peer educators.	Strongly agree	100	100	Consensus achieved
6. Editorial independence	The strategies balance stakeholder input with evidence-based practices.	Strongly agree	100	100	Consensus achieved
	The strategies maintain objectivity in their content and recommendations.	Strongly agree	100	100	Consensus achieved
	Strategies are free from potential biases, conflicts of interest, or undue influence from external parties.	Strongly agree	100	100	Consensus achieved

Note: The column “Pursue to Round 2” was used in Round 1 to indicate progression of items. As all items had already advanced by Round 2, the focus shifted to refinement and consensus validation, and the column was therefore omitted in Table 3.

### Analysis of Round 2 results

The second round of the Delphi process demonstrated a high degree of consensus across all six evaluated domains, indicating significant refinement and convergence of expert opinion, following the revisions made after the first round.

In the domain of scope and purpose, consensus was fully achieved, with 100% of participants strongly agreeing that the strategies for peer education were clearly defined and aligned with institutional goals. The strong level of agreement highlights the clear articulation and alignment of the foundational intent and strategic orientation with institutional priorities.

Under rigour, participants unanimously endorsed the alignment of objectives with the priorities of target groups (i.e., students and peer educators), and 90% confirmed the incorporation of evidence-based practices into the framework’s design. This reflects growing confidence among experts in the methodological soundness and empirical grounding of the strategies.

In the domain of stakeholder involvement, consensus was also achieved, with 90% – 100% agreement on items related to inclusivity and responsiveness to the needs of marginalised student populations. The results underscore the framework’s strength in promoting representational equity and stakeholder engagement, essential attributes for sustainable peer education initiatives.

The domain of clarity and presentation received similarly strong endorsement, with 90% – 100% agreement on the articulation of roles within the framework. This suggests that the revisions implemented post-Round One successfully enhanced the communicative clarity and structural transparency of the strategies.

For applicability, all items achieved full consensus, with strong agreement that the strategies are practical, adaptable across contexts, and responsive to current challenges faced by peer educators and students. These findings reinforce the perceived operational viability of the model within diverse higher education settings.

The qualitative feedback obtained from the Delphi rounds was subjected to thematic analysis, wherein responses were systematically coded and categorised to identify common patterns, suggestions and concerns expressed by the expert panel (Hsu & Sandford 2007; Jacob et al. 2018). These themes directly informed the iterative process of refining the statement related to the strategies of peer education. To ensure data integrity, a data cleaning process was undertaken, which involved reviewing for missing entries and assessing internal consistency between Likert-scale responses and accompanying qualitative comments. This rigorous procedure enhanced the credibility of the findings by ensuring that revisions to the strategies were firmly grounded in both empirical evidence and expert consensus (Hasson & Keeney 2021; Helms et al. 2016). The multiphased qualitative data facilitated a nuanced and comprehensive interpretation of expert perspectives, thereby supporting the construction of methodologically robust and contextually relevant peer education strategy (Carolan et al. 2022; Chan et al. 2020).

Overall, the results of Round 2 reflect a robust alignment of expert perspectives across all domains, indicating that the revised peer education strategies are methodologically sound, contextually relevant and broadly acceptable to stakeholders. These validation findings underscore the strategies’ credibility and practical feasibility, as well as their alignment with the evolving needs of peer education within higher education contexts. By triangulating data from phenomenographic analysis and an integrative review of relevant literature, and further validating the framework through expert consensus, the study presents a comprehensive and methodologically rigorous approach to the development of impactful and context-sensitive educational strategies.

**FIGURE 1:**
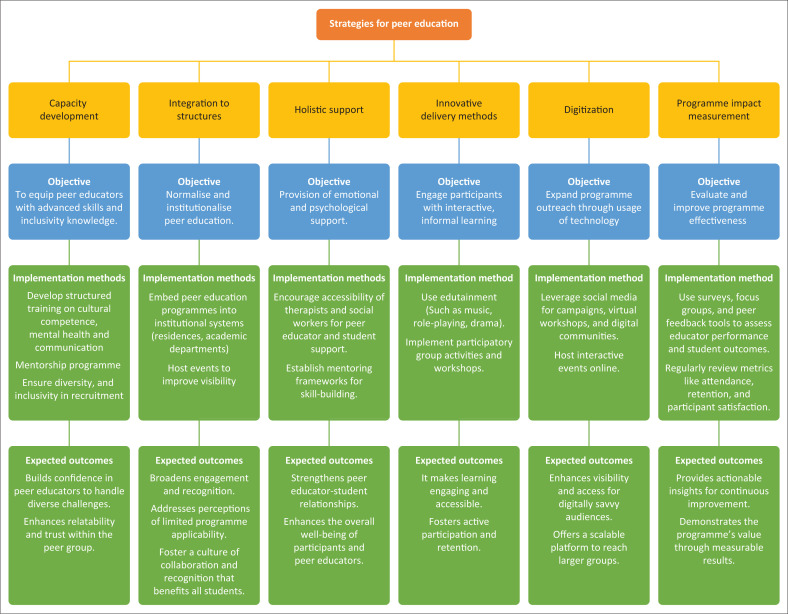
Strategies for peer education programme.

Figure 1, outlines a comprehensive set of strategies systematically developed, refined and validated through the Delphi method. This method ensured that the proposed strategies were not only theoretically sound but also practically relevant, contextually applicable and designed to enhance the implementation of the peer education programme.

### Measures of trustworthiness

The study’s trustworthiness and rigour were ensured through Delphi best practices. Credibility was upheld by engaging a diverse expert panel, iterative consensus rounds and expert validation of strategies. Triangulation combined empirical findings with integrative literature reviews and scholarly insights. Transferability was supported by thorough documentation for replication. Dependability arose from systematic scoring rounds, and confirmability was affirmed through a final expert review, ensuring accuracy and alignment with study objectives.

## Discussion

This study sought to validate peer education strategies suitable for higher education through a triangulated methodological approach that combined phenomenographic analysis, a comprehensive literature review and a modified Delphi process. The findings from Round 2 of the Delphi process revealed strong expert consensus across various strategic domains, including clarity, applicability, stakeholder involvement and rigour, affirming the framework’s methodological integrity, contextual relevance and practical feasibility. The subsequent discussion critically evaluates the significance of these strategies, situating them within the existing body of literature and exploring their theoretical and practical implications for peer education in higher education contexts.

These findings align with current scholarly discussions regarding peer education’s role in higher education. The literature review identified crucial thematic areas that shaped the strategy development, with studies emphasising the role of peer education in fostering personal and professional growth through enhanced leadership and communication skills (Nkurunziza, Uwizeye & Mukeshimana 2024a; Southgate & Aggleton 2017b; Weston 2018b). The Delphi panel’s consensus on the strategies’ clarity and alignment with institutional goals reaffirms the central role of peer programmes in promoting both academic and psychosocial competencies among students (Saiz, Gómez & Boud 2020).

The literature also highlights challenges in implementing these strategies, such as recruitment issues, ethical dilemmas and concerns regarding fidelity of delivery (Gobbo et al. 2023; Nygren & Carlson 2017). These challenges informed the assessed strategy domains within the Delphi process, particularly those emphasising stakeholder involvement and rigour. High consensus in Round 2 suggests that the revised strategies adequately address these concerns, integrating structural clarity and ethical guidance (Supatah, Fitriani & Thohri 2024).

Moreover, innovative engagement tools such as digital storytelling and online platforms have been cited as essential for effectively reaching today’s student populations (Choorat, Suksaroj & Thipayasotorn 2018b; Salzman 2014; Zhao 2024). These insights informed the creation of strategies designed to ensure adaptability and responsiveness to changing institutional contexts, elements that were fully supported during Delphi validation (Boulkedid et al., 2021).

The theoretical foundations guiding this study are firmly rooted in frameworks such as Participatory Action Research (PAR), which emphasises collaboration in strategy development and stakeholder empowerment (Nuttall et al. 2022). The iterative feedback across Delphi rounds reinforced expert engagement and ensured the reflective, practical and theoretical interventions of strategies. Furthermore, Rogers’ Diffusion of Innovations Theory accentuates the significance of the strategies’ clear advantages and compatibility with existing values, as reflected by the consensus achieved during the Delphi process (Dwangu & Mahlangu 2021).

The iterative refinement process not only clarified ambiguous terminology but also manifested the principles of Transformative Learning Theory, which focuses on critical reflection and dialogic exchange (Aristovnik et al. 2020). The evolving expert interpretations and the continuous consolidation of strategies across rounds illustrate the transformative potential inherent in consensus-building within peer education.

Finally, the results resonate with Constructivist Theory, which posits that knowledge is constructed through social interaction (Chin et al. 2024; Utvaer et al. 2022). The synthesis of the phenomenographic insights, extensive literature evidence and expert validation exemplifies this constructivist approach, grounding the developed strategies in shared understanding and practical relevance. Overall, the Delphi validation process, underpinned by theoretical constructs and empirical insights, lends robust credibility and potential utility to the peer education strategy, indicating a significant step in enhancing peer-led initiatives within higher education.

### Recommendations

The modified Delphi approach has emerged as an effective and rigorous method for validating peer education strategies, offering broad applicability across diverse educational contexts. Through iterative consensus-building among experts from institutions situated in varied geographic locations, the Delphi process has facilitated the systematic validation of these strategies. The outcomes indicate the potential of the validated strategies to significantly enhance the design and implementation of peer education programmes. Moreover, this approach establishes a solid foundation for the institutionalisation of peer education, supported by mechanisms for ongoing feedback and evaluation that promote continuous refinement and ensure alignment with the evolving needs of students and institutional objectives.

### Strengths and limitations

The study’s strengths include its rigorous methodological approach, expert-driven validation and applicability to diverse institutional settings. Limitations include a relatively small expert panel and the focus on institutions within a specific geographic region, which may limit generalisability.

## Conclusion

This study presents a rigorously validated strategy framework for peer education in the context of higher education, grounded in empirical evidence and expert consensus. The strategies address critical dimensions including purpose clarity, methodological rigour, stakeholder inclusivity, role clarity, applicability and editorial independence. Their validation through the Delphi method ensures their relevance, feasibility and adaptability. The findings offer higher education institutions a practical guide to designing, implementing and institutionalising effective peer education programmes. These findings further provide an opportunity for further research to test the long-term outcomes of these strategies across diverse academic environments, further enhancing their credibility and contribution to student wellness and academic success.
